# Preventive Effects of Collagen Peptide from Deer Sinew on Bone Loss in Ovariectomized Rats

**DOI:** 10.1155/2014/627285

**Published:** 2014-07-01

**Authors:** He Zhang, Ying Dong, Bin Qi, Li Liu, Guangxin Zhou, Xueyuan Bai, Chunhui Yang, Daqing Zhao, Yu Zhao

**Affiliations:** ^1^Changchun University of Chinese Medicine, Boshuo Road 1035, Changchun, Jilin 130117, China; ^2^The Affiliated Hospital to Changchun University of Chinese Medicine, Gongnong Road 1478, Changchun, Jilin 130021, China

## Abstract

Deer sinew (DS) has been used traditionally for various illnesses, and the major active constituent is collagen. In this study, we assessed the effects of collagen peptide from DS on bone loss in the ovariectomized rats. Wister female rats were randomly divided into six groups as follows: sham-operated (SHAM), ovariectomized control (OVX), OVX given 1.0 mg/kg/week nylestriol (OVX + N), OVX given 0.4 g/kg/day collagen peptide (OVX + H), OVX given 0.2 g/kg/day collagen peptide (OXV + M), and OVX given 0.1 g/kg/day collagen peptide (OXV + L), respectively. After 13 weeks of treatment, the rats were euthanized, and the effects of collagen peptide on body weight, uterine weight, bone mineral density (BMD), serum biochemical indicators, bone histomorphometry, and bone mechanics were observed. The data showed that BMD and concentration of serum hydroxyproline were significantly increased and the levels of serum calcium, phosphorus, and alkaline phosphatase were decreased. Besides, histomorphometric parameters and mechanical indicators were improved. However, collagen peptide of DS has no effect on estradiol level, body weight, and uterine weight. Therefore, these results suggest that the collagen peptide supplementation may also prevent and treat bone loss.

## 1. Introduction

Osteoporosis (OP) is a silent and potentially disabling skeletal disease. Postmenopausal women are at greatest risk for developing OP. The hallmark of menopause is a reduction in bone mass caused by an imbalance between bone resorption and formation due to loss of ovarian function [1]. The pathogenesis of postmenopausal osteoporosis involves the interplay of many factors, such as aging, genetic, environmental, hormonal, nutritional, and life style factors [2]. The more widely OP medications include estrogen, calcitonin [3], bisphosphonate [4], calcium [5], vitamin D_3_, and selective estrogen receptor modulators (SERMs) [6]; however, some medications of them have serious side effects [7]. Therefore, the interest to find effective and safe alternatives for the treatment of osteoporosis has grown in recent years.

Traditional Chinese medicines contain a number of active constituents, which have been used for centuries to treat bone disease. Because Chinese medicines have less side effects, which seem to be more suitable for a long-term medication, now Chinese researchers have studied the therapeutic effects on osteoporosis by traditional medicines which contain soy [8], epimedium [9], cortex eucommiae [10], radix dipsaci [11], fructus psoraleae [12],* Rehmannia glutinosa* [13, 14],* Astragalus membranaceus *[15], and so on.

Deer sinew (DS) is derived from limbs of* Cervus nippon* Temminck or* Cervus elaphus* Linnaeus, distributing mainly in China, Japan, and Russia. DS is one of the best known and highly valued food and medicine. According to* Chinese Materia Medica*, DS possesses the function of strengthening tendons and bones, nourishing the liver and kidney, benefiting yang, and expelling wind damp, for the treatment of arms and legs weakness, pain of waist and knees, and twitch; consumptive disease, dizziness, and epicophosis; cold intolerance; rheumatism arthritis joints pain, and so on. DS contains rich collagen with a content of 82.12% [16]. Collagen constitutes a family of proteins presenting in the extracellular matrices, taking up about 25% to 35% of the whole-body protein content; unfortunately, our body loses collagen at a rate of 1% per year, starting at the age of 25 [17]. It is ubiquitous that collagen is responsible for maintaining the structural integrity of vertebrates and many other multicellular organisms [18], such as skin, tendons, ligaments, and cartilage. The collagen peptides are extensive used as health food in Chinese drugstore. Some researchers reported that collagen peptides supplementation could strengthen skin elasticity [19], improve the bone density [20, 21], and alleviate some arthritis symptoms [22].

According to traditional Chinese medicine, collagen peptide is essential ingredients of DS and plays important roles in improving symptoms of the menopausal osteoporosis. Therefore, this study discusses whether the collagen peptide of DS prevents bone loss in ovariectomized rats.

## 2. Methods

### 2.1. Preparation of Collagen Peptide

Deer sinew (DS)* of Cervus elaphus* Linnaeus was purchased from a deer farm in Shuang Yang, Ji Lin, in November, 2010. As in previous studies the collagen peptide was obtained from DS [16]. The DS was made by steeping in 0.2% hydrochloric acid when material/liquid ratio was 1 : 12 (w/v) for 48 h at room temperature. Then collagen of DS was extracted in boiling water, adjusted to pH 7.5 with NaOH (0.5 mol/L). After adding trypsin (Beijing dingguo biotech Co., Ltd) to collagen solution (enzyme : collagen = 1 : 1000, w/w), the reaction mixture was incubated at 37°C for 3 h. To stop the reaction, the mixture was heated immediately at 100°C for 20 min and then centrifuged at 4000 ×g for 30 min, and the supernatant was freeze-dried. The powder of the water-solubilized extract was collagen peptide. The molecular weight of collagen peptide was assayed with HPLC, retention times of collagen peptide using Zenix SEC-100 (7.8 × 300 mm, 3 *μ*m) column for analysis. Elution buffer, 0.1 M phosphate buffer containing 0.1 M Na_2_SO_4_, pH6.8, flow rate was 0.5 mL/min and detection wavelength was 230 nm. The filtration calibration kits (LMW and HMW) were purchased from GE Healthcare. Protein concentration was measured by Lowry method using a protein assay kit (Beijing dingguo biotech Co., Ltd) that was around 72.25%.

### 2.2. Amino Acid Analysis

Collagen peptide was hydrolyzed under vacuum in 6 mol/L HCl for 24 h at 110 ± 1°C for general amino acid analysis. Amino acids converted to phenyl isothiocyanate (PITC) derivatives were analyzed by high performance liquid chromatography (HPLC) (Agilent 1100 series; Agilent Technologies, USA) with Wondasil-C18 (4.6 × 150 mm, 5 *μ*m). Eluents were 97% aqueous acetonitrile containing 0.1 mol/L sodium acetate (pH6.5, eluent A) and 80% aqueous acetonitrile (eluent B); flow rate was 1.0 mL/min; the detection wavelengths were at 254 nm and the column temperature was 37°C. The gradient elution procedures are ignored.

### 2.3. Animals and Treatments

Sixty female Wister rats which aged 10 weeks (210 ± 20 g) were purchased by Basic Medical Experimental Animal Center of Jilin University (Jilin, China, SCXK-JI-2008-0005) and acclimated to conditions for 1 week before the experiment. The rats were housed in an air-conditioned room with 12-hour light-dark illumination cycles and given free access to deionized water and food (commercial laboratory rat's food containing 22.0% protein, 7.90% fat, 3.0% fiber, 1.19% calcium, and 0.86% phosphorus) (Beijing Keaoxieli Fodder Co. Ltd, Beijing, China). At 11 weeks of age, the animals were either bilateral ovariectomized or sham operated (SHAM). One week after surgery, they were equally divided into six groups: SHAM, ovariectomized control (OVX), OVX given 1.0 mg/kg/week nylestriol (OVX + N) (nylestriol tables were purchased from Shanghai New Hualian Pharmaceutical Co., Ltd, Shanghai, China), OVX given 0.4 g/kg/day collagen peptide (OVX + H), OVX given 0.2 g/kg/day collagen peptide (OXV + M), and OVX given 0.1 g/kg/day collagen peptide (OXV + L), respectively. The SHAM and OVX were given oral gavages of deionized water. The nylestriol tables and collagen peptide were dissolved in deionized water. Then, they were given to the rats via oral gavage. The nylestriol group acted as positive control. These rats were administrated for 13 weeks. The body weight of each animal was measured weekly, and the dosage of drug administered was calculated based on the most recent body weight measurement. This experiment was approved by the Bioethics Committee of the Chang Chun University of Chinese Medicine, and the procedures of the experiment strictly adhered to generally accepted international rules and regulations.

### 2.4. Sample Collection

After 13 weeks of drug treatment, the experimental rats were fasted for 24 h and anesthetized. Blood samples were collected from the abdominal artery and centrifuged at 6000 ×g for 10 min; then, serum samples were stored at −80°C for biochemical determinations. The left femurs were dissected, then placed in physiologic saline, and stored at −20°C for measurement of bone mineral density (BMD) and bone mechanics indicators [23]. The right tibia and lumbar vertebrae were removed and fixed in 10% neutral-buffered formalin for measuring of bone histomorphometry parameters. The uterus was removed from each rat, cleaned of adhering soft tissues, and immediately weighed.

### 2.5. Serum Biochemical Analysis

The concentrations of serum calcium (Ca) and phosphorus (P), alkaline phosphatase (ALP), and hydroxyproline (Hyp) were measured on an automatic chemistry analyzer (OLYMPUS 2700, Japan) using determination kit. The levels of serum estradiol (E_2_) were measured by ELISA kit. These kits were purchased from BioSino Bio-technology and Science Inc. (Beijing, China).

### 2.6. Bone Mineral Density Analysis

The left femur was thawed for 12 h before the experiments and kept wrapped in gauze soaked with a 0.9% saline solution. The bone mineral density (BMD, cm^2^) of the distal one third of left femur was measured by single-energy X-ray densitometry (Model HH6005, Beijing Hehai Advanced Technology Co., Ltd., China).

### 2.7. Bone Mechanics Indicators Evaluation

The bone mechanics indicators of left femur were assessed by using a universal testing machine (Model AG-107A, Shimadzu, Japan). The fulcrum span of experimental parameter was 20 mm; the loading speed was 2 mm/min. The test program interrupted the test at the specimen's breaking point. The stress, strain, and load parameters were recorded by the software. The maximum load (*L*_max⁡_), maximum deflection (*D*_max⁡_), maximum bending moment (*B*_max⁡_), and bone stress (*σ*) were calculated.

### 2.8. Bone Histomorphometric Studies

The right tibia and lumbar vertebrae were fixed in 10% neutral formalin solution followed by decalcification with formic acid, dehydrated in increasing gradients of alcohol, cleared with xylene, and embedded in paraffin. The paraffin embedded tissues were cut into 5-micron sections with a microtome. Hematoxylin and eosin staining were performed and observed by light microscope (Model BX51, OLYPUS, Japan). Histomorphometry of the trabecular bone of tibia and lumbar vertebra was done by using an image analysis system. The parameters measured in this study were trabecular bone volume, trabecular thickness, trabecular number, and trabecular separation. Trabecular numbers (Tb.N, *n*), trabecular thickness (Tb.Th, *μ*m), trabecular separation (Tb.Sp, *μ*m), trabecular bone volume (BV/TV, %), and cortical bone thickness (Cb.Th, *μ*m) were calculated [24].

### 2.9. Statistical Analysis

The results were presented as mean ± SD, and one-way analysis of variance followed by Dunnett's* t*-test were used for statistical analysis (PASW 19.0 software; SPSS Inc., Chicago, USA), and the significance level was set at *P* < 0.05 (*), *P* < 0.01 (**), and *P* < 0.001 (***) for all tests.

## 3. Results

### 3.1. Molecular Weight and Amino Acid Contents

**Figure 1:**
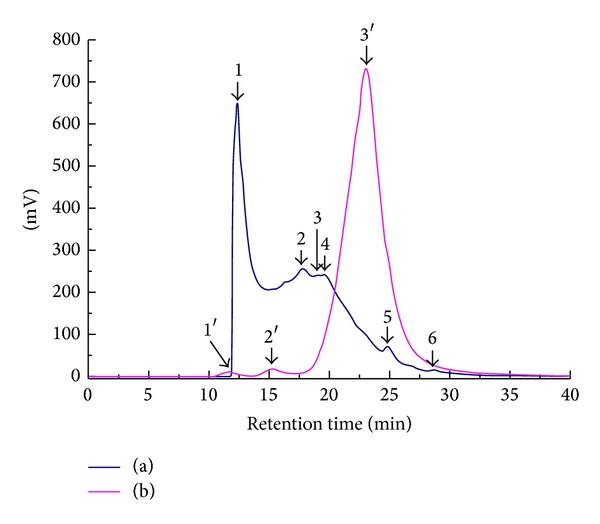
HPLC profiles of collagen peptide of deer sinew. Note: (a) collagen of deer sinew was extracted in boiling water. (b) Collagen peptide was treated with trypsin (1 : 1000, w/w) at 37°C for 3 h after boiling water.

HPLC gel filtration chromatography was used to evaluate the molecular weight. HPLC profile of collagen of deer sinew (DS) was shown in Figure 1(a); HPLC profiles of collagen peptide of DS were shown in Figure 1(b). There was a linear relation between the retention time and the log of the molecular mass of the reference proteins in the range of 669000–3323 Da (*y* = −0.1840*x* + 7.8414, *r* ^2^ = 0.9934). Molecular size distributions with HPLC of collagen peptide were showed in Table 1. Collagen of DS was mainly distributed between 17,000 and 367,000 Da. After trypsin enzymolysis for 3 h, about 98% of the total collagen peptide was about the 4,000 Da.

As shown in Table 2, the collagen peptide contained seven essential amino acids: lysine, leucine, phenylalanine, threonine, valine, isoleucine, and methionine. Total content of the hydrophobic amino acids was 38.28%. Glycine (32.81%) was the major component in the amino acids of collagen peptide. Proline and hydroxyproline accounted for 14.59% and 6.99% of the total amino acid residues, respectively. Tyrosine, methionine, and histidine were lowest in the amino acids of collagen peptide.

### 3.2. Bone Mineral Density

**Figure 2:**
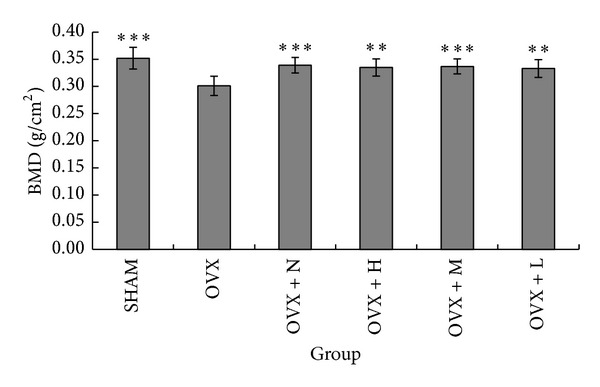
BMD for all the groups after 13 weeks of treatment. Note: **P < 0.01 and ***P < 0.001 compared with OVX group.

Bone loss was evident at the femur sites in ovariectomized control (OVX) mice compared with age matched sham-operated (SHAM) at the end of the study. The results showed that the bone mineral density (BMD) of femur was significantly decreased by 14.49% in OVX (0.301 ± 0.018) as compared with the SHAM (0.352 ± 0.020) (Figure 2). The results showed that the administration of collagen peptide had antiosteoporotic effect, and OVX + H, OVX + M, and OVX + L groups significantly recovered the BMD of OVX rats by 10.03%, 10.75%, and 9.50%, respectively.

### 3.3. Body and Uterine Weight

**Figure 3:**
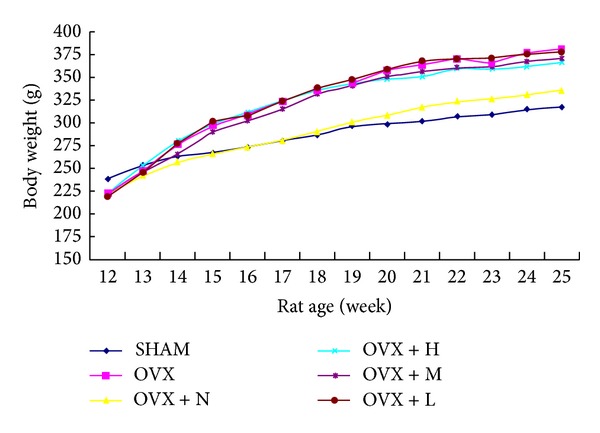
The changes in body weight in each group during 13 weeks.

**Figure 4:**
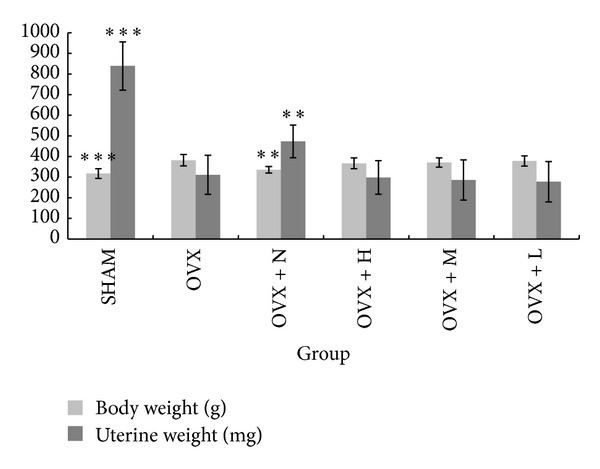
Body and uterine weight in different treatment groups after 13 weeks. Note: **P < 0.01 and ***P < 0.001 compared with OVX group.

The changes in the body weights of the rats in each group were shown in Figure 3. After surgery, four groups of rats had a similar initial mean body weight. The administration of collagen peptide and OVX groups had significantly greater weight gain than rats in the SHAM and OVX + N after the age of 25 weeks. As shown in Figure 4, at the end of the experiments, the body weight of rats in the OVX group (381.8 ± 27.96) was 20.33% higher than that of the SHAM rats (317.3 ± 23.95). The administration of collagen peptide (0.4, 0.2, and 0.1 g/kg/day) could not prevent the increase of body weight associated with E_2_ deficiency, while the nylestriol (1.0 mg/kg/week) could significantly prevent weight gain. The uterine weight of OVX rats was significantly decreased relative to SHAM (Figure 4), indicating the success of the surgical procedure. Collagen peptide (0.4, 0.2, and 0.1 g/kg/day) treatment did not affect the uterine weight of OVX rats, while uterine weight of the OVX + N group was significantly increased against those of OVX group.

### 3.4. Serum Biochemical Marker

Table 3 showed ovariectomy-induced significant changes in the serum biochemical marker. Compared with SHAM rats, OVX for 13 weeks significantly increased serum calcium (Ca) by 7.63%, phosphorus (P) by 14.10%, alkaline phosphatase (ALP) by 31.72%, hydroxyproline (Hyp) by 8.91%, and reduced serum estradiol (E_2_) by 61.95%. Compared with OVX group, the collagen peptide (0.4 g/kg/day) significantly decreased levels of serum Ca, P, and ALP; the collagen peptide (0.4, 0.2, and 0.1 g/kg/day) did not affect the levels of serum E_2_, while nylestriol (1.0 mg/kg/week) significantly increased the levels of serum E_2_ by 59.98%; the contents of Hyp were remarkably increased in the administration of collagen peptide, because the collagen peptide was hydrolyzed to break down into its compont parts in the body. The results showed that the high (0.4 g/kg/week) dose of collagen peptide had marked improvement for most of serum biochemical markers.

### 3.5. Bone Mechanics Indicators

As shown in Table 4, the maximum load (*L*_max⁡_), maximum deflection (*D*_max⁡_), maximum bending moment (*B*_max⁡_), and bone stress (*σ*) were significantly decreased by 30.56, 31.65, 30.56, and 22.76%, respectively, in OVX as compared with SHAM. Compared with OVX group, the OVX + H group significantly increased *L*_max⁡_, *D*_max⁡_, and *B*_max⁡_; the OVX + M group increased *L*_max⁡_ and *B*_max⁡_. The collagen peptide (0.4, 0.2, and 0.1 g/kg/day) treatment did not affect the σ of OVX rats. The aforementioned results indicated that collagen peptide (0.4 g/kg/day) could significantly improve the bone mechanics indicators of OVX groups.

### 3.6. Bone Histomorphometric Parameters

**Figure 5:**
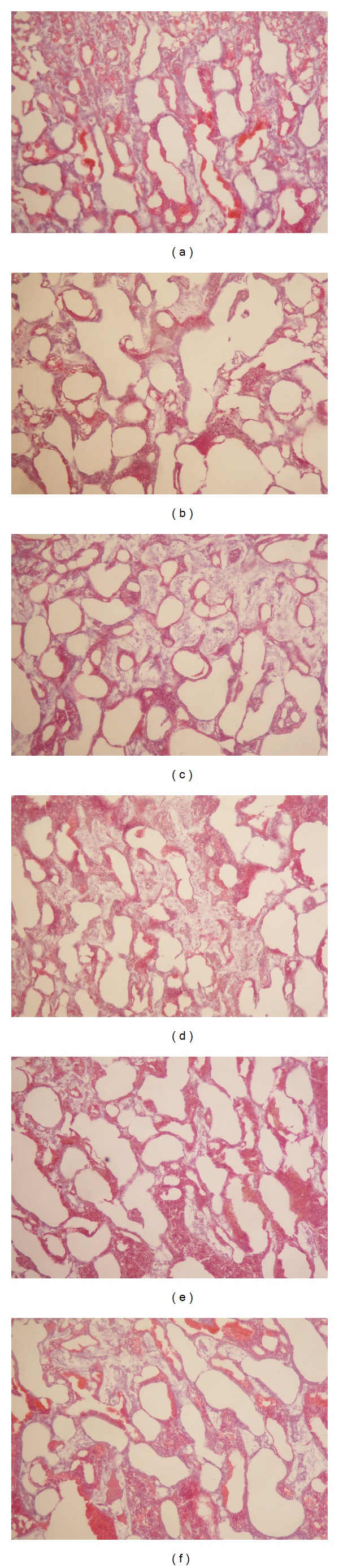
Photomicrographs of trabecular bone. Note: undecalcified section (100x magnification) shows trabecular bone using multifunction true color pathological image analyzer. (a) SHAM, (b) OVX, (c) OVX + N, (d) OVX + H, (e) OVX + M, and (f) OVX + L.

Table 5 and Figure 5 showed significant changes in the femur microstructure and parameters. Compared with SHAM rats, OVX significantly reduced trabecular numbers (Tb.N) by 75%, trabecular thickness (Tb.Th) by 3.52%, trabecular bone volume (TV/BV) by 44.57%, and cortical bone thickness (Cb.Th) by 2.13% and increased trabecular separation (Tb.Sp) by 73.05%. By administering collagen peptide (0.4, 0.2, and 0.1 g/kg/day), the Tb.N, Tb.Th, TV/BV, and Cb.Th were increased significantly, but Tb.Sp was markedly reduced compared to OVX groups.

## 4. Discussion

The ovariectomized (OVX) rat is a well-known osteoporosis model, because the mechanism of controlling the gain and loss of bone mass is similar to human. Bone mineral density (BMD) is considered to be the standard measure for the diagnosis of osteoporosis and the assessment of fracture risk, which can respond to the bone mass. Postmenopausal bone loss is characterized by a decrease in BMD and a microarchitecture deterioration of trabecular bone, with a particular diminution in the total number of trabecular and an increase in the number of their perforations [25]. Shuster [17] reported that a loss of collagen in skin and bones with aging was the causal counterpart to loss of bone density in senile osteoporosis. According to results of experiment, the collagen peptide supplement significantly increased the BMD of the femur in ovariectomized rats (Figure 2). The trabecular numbers (Tb.N), trabecular thickness (Tb.Th), cortical bone thickness (Cb.Th), and trabecular bone volume (BV/TV) were increased, while trabecular separation (Tb.Sp) was decreased in the OVX + H group (Table 5). These results show that collagen peptide supplement can reduce the bone turnover and improve the trabecular bone microarchitecture.

A marked atrophy of the uterus has been used as evidence of the success of ovariectomy, and it has been reported that estrogen deficiency following ovariectomy increased body weight [26], which had been postulated to be associated with regulatory effect of estrogen on the adipose tissue [27]. Our experimental results indicated that ovariectomy also led to a dramatic decrease in uterine weight (Figure 4) and levels of serum estradiol (E_2_) (Table 3) and increase in body weight (Figure 4), while the administration of collagen peptide did not reverse the changes. So, it is suggested that the collagen peptide does not influence the actions of estrogen or its receptor.

Calcium (Ca) and phosphorus (P) are required for normal skeletal growth and mineralization and play an important role in regulating bone remodeling and bone mass. Ca is a naturally occurring hormone that acts to inhibit bone resorption by decreasing osteoclast activity [7]. P or acidic amino acids contained in collagen peptide could selectively bind calcium and facilitate Ca transports across the intestinal membranes [28]. Ovariectomy-related bone loss also decreased contents of bone Ca and P and increased contents of serum Ca and P, while the administration of collagen peptide (0.4 g/kg/day) decreased levels of serum Ca and P against that of the OVX (Table 3). We speculate that collagen peptide supplement could promote collagen synthesis and inhibit loss of Ca and P. ALP is the biochemical marker of bone turnover, affects osteoid formation and mineralization, and is still the most widely used in clinical practice [29]. During bone resorption, highly active osteoclasts may secrete factors into the space between the cell and bone surface such as acid, matrix metalloproteinases (MMPS), and cathepsin K in excess that can degrade collagen type I into Hyp [1]. Our results supported the notion that, compared with SHAM group, the content of Hyp significantly increased in the OVX group, because the collagen peptide is hydrolyzed to break down into Hyp in the body.

Whole-bone stiffness and strength show a function of total bone mass, the tissue geometric distribution, and material properties [30]. The estrogenic deficit caused by bilateral oophorectomy resulted in a loss of bone mass, with a consequent decrease in bone mechanics indicators (Table 4), and the reduced concentration of bone collagen reducible cross-links also was associated with reduced bone strength [31, 32]. Our experimental results show that collagen peptide can enhance the bone biomechanical properties of ovariectomized rats.

Bone is a living dynamic metabolic system which is made up of approximately 70% inorganic salts and 30% organic matrix by weight; however, collagen (mostly type I collagen) makes up over 90% of the organic component. It is actually collagen that generates the bone density that gives our bones the ability to withstand stress. Collagen is a protein that provides a framework of soft tissue which calcium adheres onto, creating a hardened framework. Calcium is still an important player, but, without enough collagen, taking large doses can be wasteful. Several studies have demonstrated that bone loss increased postmenopausal osteoporosis, the resorption of the organic matrix over formation. The decreased rate of collagen synthesis may also lead to a change in the quality of collagen fibers, which weakens them. After menopause, hormonal and systemic factors may also directly or indirectly modify type I collagen properties, such as collagen degradation [31], overhydroxylation, and poor cross-linking [33, 34]. So, collagen plays an important role in therapy of osteoporosis. Deer sinew (DS) is widely used for many years in China to strengthen tendons and bones. Sun et al. [22] reported that collagen isolated from DS ameliorated Freund's adjuvant (CFA) induced adjuvant arthritis by reducing serum IL-l*β* and TNF-a inflammatory cytokines. Collagen of DS prevented retinoic acid induced osteoporosis by increasing BMD and improving bone mechanics indicators and bone histomorphometric parameters [21]. Our experiments show that administration of collagen peptide of DS significantly affects the skeletal system of ovariectomized rats, counteracts some changes induced by estrogen deficiency, and also induced changes which seem to be unrelated to typical estrogenic effects in rats with decreased estrogen levels. Although collagen peptide has been considered as therapeutic agent of treat bone diseases, it remains unknown whether collagen peptide modulates bone metabolism or not.

The sequence of collagen is predominantly repeats of the Gly-X-Y triplet, where X can be any other amino acid but is usually a proline, and Y is often a hydroxyproline. Collagen is constituted by three polypeptide chains that form a triple helix structure. The average molecular weight of one chain amounts to about 90 kDa. Our results showed that content of amino acid of collagen peptide complied with the amino acid composition (Table 2). Because of large molecular weight, collagen is difficult to be absorbed by body. Enzymatic hydrolysis of collagen is one approach used to release bioactive protein and is widely applied to improve functional and nutritional properties of collagen sources. The researches showed that collagen peptides of less than 3000 Da molecular weight could be readily absorbed by gastrointestinal tract [35]. It appeared in the human blood party in a small peptide form [36, 37] and was accumulated in skin for up to 96 h [38]. Collagen peptides have been reported to perform strong antioxidant activities [39, 40]. However, low molecular weight collagen peptide (about 4000 Da) from DS accounted for 98% (Table 1). Collagen hydrolysate has long been used in pharmaceuticals and food supplements for improving skin and bone tissues [19]. Administering shark skin gelatin to the ovariectomized rats resulted in the bone mineral density of the femur epiphysis being higher than that in the sham-operated rats. The contents of type I collagen and glycosaminoglycan in the epiphysis were increased by administering shark skin gelatin [41]. Han et al. [42] reported that the cod bone gelatin treatment prevented bone loss by decreasing bone resorption in OVX rats with established osteopenia and it might also exert its action through modulation of RANKL and OPG expression and suppression of proinflammatory cytokines release, which in turn were important for the consequent osteoclastogenesis. The vertebrae of the ovariectomized group that received the higher dosage of collagen hydrolysates withstood a load four times greater and exhibited higher levels of protein and osteocalcin content than those receiving either gelatin or no supplement, and, at the same time, these results supported the notion that supplementing the normal diet of castrated female rats with 10 times the amount of CH suggested for humans can have a protective effect on the loss of mass, total protein, and physical strength of the rodent's bone system [43]. These studies have shown that gelatin hydrolysate or collagen peptides may be effective in minimizing bone loss in OVX rats.

As a conclusion, collagen peptide from deer sinew can inhibit the progression of bone loss induced by ovariectomy in rats. The potential antiosteoporotic mechanism conjectured that collagen peptide provides a plenty of glycine, proline, and other amino acids to accelerate collagen synthesis, and collagen peptide has the antioxidative activity to reduce the intercellular free radicals to protect the osteoblasts. We believe that collagen peptide may be useful in human for the prevention of not only postmenopausal osteoporosis but also osteoporosis in general. Further studies are required to determine its antiosteoporotic mechanism of action for the prevention and treatment of osteoporosis.

## Supplementary Material

The graphical abstract is summarized the contents of the paper in a concise.

## Figures and Tables

**Table 1:** Molecular size distributions with HPLC of collagen peptide.

Number	Retention time (min)	Molecular weight (Da)	Peak area percentage (%)	Number	Retention time (min)	Molecular weight (Da)	Peak area percentage (%)
1	12.37	367553	35.38	1′	12.31	377016	0.59
2	17.80	36829	29.13	2′	15.29	106669	1.09
3	19.07	21504	5.26	3′	23.05	3982	98.32
4	19.58	17325	24.99				
5	24.80	1897	4.38				
6	28.62	376	0.86				

**Table 2:** Amino acid composition of collagen peptide.

Amino acid	Content (%)
Gly	32.81
Ala	14.80
Pro	14.59
Hyp	6.99
Arg	6.94
Lys	5.47
Glu	3.18
Leu	2.97
Phe	2.47
Asp	2.40
Ser	2.14
Val	1.88
Thr	1.53
Ile	0.87
Tyr	0.37
Met	0.33
His	0.28

**Table 3:** Effects of collagen peptide on serum biochemical markers in ovariectomized rats (*n* = 10).

Group	Target				
	Ca (mmol/L)	P (mmol/L)	ALP (U/L)	E_2_ (pg/mL)	Hyp (*µ*g/mL)
Sham	2.36 ± 0.13**	1.56 ± 0.12**	82.43 ± 12.47**	26.99 ± 6.08***	28.86 ± 2.19*
OVX	2.54 ± 0.11	1.78 ± 0.15	108.58 ± 16.96	10.27 ± 2.65	31.43 ± 1.78
OVX + N	2.43 ± 0.12*	1.65 ± 0.11*	93.64 ± 10.38*	16.43 ± 3.62**	29.75 ± 1.98
OVX + H	2.43 ± 0.11	1.65 ± 0.07*	94.68 ± 11.35*	10.06 ± 3.54	37.43 ± 2.96***
OVX + M	2.41 ± 0.14	1.70 ± 0.09	97.33 ± 10.68	10.31 ± 4.05	36.68 ± 3.29**
OVX + L	2.45 ± 0.10	1.69 ± 0.15	94.22 ± 15.53	10.04 ± 4.06	34.38 ± 4.14

Note: *P < 0.05, **P < 0.01, and ***P < 0.001 compared with OVX group.

**Table 4:** Effects of collagen peptide on bone mechanics indicators in ovariectomized rats (*n* = 10).

Group	Target			
	*L*_max⁡_ (N)	*D*_max⁡_ (mm)	*B*_max⁡_ (N)	*σ* (N/mm^2^)
Sham	132.20 ± 26.49**	0.79 ± 0.26*	661.00 ± 132.47**	123.90 ± 23.17*
OVX	91.80 ± 17.99	0.54 ± 0.17	459.00 ± 89.93	95.70 ± 23.00
OVX + N	121.80 ± 8.99**	0.73 ± 0.23	609.00 ± 44.96**	137.68 ± 27.51*
OVX + H	114.60 ± 16.08*	0.71 ± 0.13*	573.000 ± 80.42*	120.28 ± 36.19
OVX + M	110.40 ± 21.63*	0.63 ± 0.19	552.00 ± 108.17*	108.60 ± 19.85
OVX + L	100.80 ± 22.33	0.58 ± 0.10	504.00 ± 111.67	109.63 ± 31.13

Note: *P < 0.05 and **P < 0.01 compared with OVX group.

**Table 5:** Effects of collagen peptide on bone histomorphometric parameters in ovariectomized rats (*n* = 10).

Group	Target				
	Tb.N (*n*)	Tb.Th (*µ*m)	Tb.Sp (*µ*m)	TV/BV (%)	Cb.Th (*µ*m)
SHAM	3.60 ± 0.70***	8.25 ± 0.16**	9.50 ± 1.03***	36.64 ± 3.27***	12.21 ± 0.14**
OVX	0.90 ± 0.32	7.96 ± 0.20	16.44 ± 1.51	20.31 ± 4.17	11.95 ± 0.11
OVX + N	1.70 ± 0.67*	8.12 ± 0.12	12.95 ± 1.92**	25.36 ± 4.58*	12.15 ± 0.21*
OVX + H	1.70 ± 0.48**	8.13 ± 0.13*	12.68 ± 0.65***	27.28 ± 1.94**	12.12 ± 0.19**
OVX + M	1.60 ± 0.52*	8.11 ± 0.17	13.09 ± 1.16***	26.58 ± 2.24**	12.11 ± 0.24
OVX + L	1.60 ± 0.52**	8.17 ± 0.17	13.27 ± 0.87***	26.36 ± 2.26**	12.14 ± 0.24

Note: *P < 0.05, **P < 0.01, and ***P < 0.001 compared with OVX group.
