# Supplementary material for: Preventive Effects of Collagen Peptide from Deer Sinew on Bone Loss in Ovariectomized Rats
Source: Evid Based Complement Alternat Med. 2014 Jul 1;2014:627285. doi: 10.1155/2014/627285 (PMC4102020; doi:10.1155/2014/627285)
Supplement: Supplementary file 1 — The graphical abstract is summarized the contents of the paper in a concise. [file 627285.f1.doc]

Graphical Abstract

The graphical abstract is summarized the contents of the paper in a concise.

*

*

Fig.1 Graphical abstract
